# Association study of gene polymorphisms and depression with abnormal humor in traditional Uighur medicine

**DOI:** 10.1186/1472-6882-13-332

**Published:** 2013-11-25

**Authors:** Abdiryim Yusup, Hanzohra Upur, Ayimgul Abla, Halmurat Upur

**Affiliations:** 1 Collage of Traditional Uighur Medicine, Xinjiang Medical University, Xinyi Road 393, Urumqi, China

**Keywords:** Traditional Uighur medicine, Depression, 5-HT_2A_, BDNF, 5-HT_1A_

## Abstract

**Background:**

According to the humor theory of Traditional Uighur Medicine (TUM), a same disease is classified into different abnormal humor types and corresponding methods are applied to treat the diseases according to the type of abnormal humor characteristics. To date the biological foundation of classification of diseases by humor theory has been little studied and the mechanism of action is still unclear. In the present study, we aimed to investigate the association between some related gene polymorphisms and depression with abnormal humor in TUM.

**Methods:**

201 cases of depression patients in a Uighur population were divided into two groups as: 107 cases of depression patients with abnormal black bile (ABB), 94 cases of depression patients with none abnormal black bile (nABB), and 50 healthy people were served as control group. Venous blood was used to isolate DNA samples, and the polymerase chain reaction-restriction fragment length polymorphism (PCR-RFLP) technique was used for genotyping of single nucleotide polymorphisms (SNPs). Polymorphisms in the serotonin 2A (5-HT_2A_) receptor gene, brain derived neurotrophic factor (BDNF), serotonin 1A (5-HT_1A_) receptor gene were investigated in each groups, respectively.

**Results:**

The 5-HT_2A_ A-1438G, 5-HT_2A_ T102C, BDNF Val66Met, and 5-HT_1A_ C-1019G gene polymorphisms showed significant association with ABB. However, no difference between nABB and controls was found for those genotype distribution and allele frequency. Moreover, the T102C and A1438G SNPs in the 5-HT_2A_ receptor gene polymorphisms were in linkage disequilibrium. In addition, the OR associated with the combination of Val66Met-Val/Val genotype plus the presence of -1019C allele was 8.393 for ABB compared with controls (OR 8.393; 95% CI 1.807 ~ 38.991; *P*= 0.003). Moreover, the OR associated with the presence of -Met plus -1019C alleles was 12.194 for ABB compared with controls (OR 12.194; 95% CI 1.433 ~ 103.776; *P*= 0.005). The OR associated with the presence of -1438C/C plus Val/Val genotypes was 7.738 for ABB compared with controls (OR 7.738; 95% CI 1.566 ~ 38.241; *P*= 0.005).

**Conclusion:**

It was concluded that there were significant relationship between the gene polymorphisms and classification of depression with abnormal humor in TUM. The 5-HT_2A_ A-1438G, 5-HT_2A_ T102C, BDNF Val66Met, and 5-HT_1A_ C-1019G gene polymorphisms might predict the incidence of depression with ABB.

## Background

Depression is a major public health problem. It tends to have a chronic course, produces disability and is associated with suicide [1,2]. There are overwhelming justifications to explore the molecular mechanisms underlying major depressive disorder [3], depression significantly complicates chronic illness [4] and depression is the leading cause of disability worldwide [5].

The disease of depression or melancholy (black bile in Greek) was linked to the Hippocratic/Galenic medical theory of the four humors. Humoral Theory was initially Greek philosophy and firstly brought to medicine by Hippocrates (c.460-370 B.C.), adopted and developed by Greek (e.g. Galen, c.129-216 A.D.), Roman and Islamic physicians (e.g. Avicenna, c.980-1037 A.D.), it became the most commonly held view of the human body among European physicians until the discoveries of the functions of the circulatory, respiratory and digestive systems in 18th century. Essentially, this theory holds that human body was filled with four basic substances or humors- blood, phlegm, yellow bile and black bile. According to Hippocrates Medicine, the four humors needed to be balanced for good health, and an excess or deficit of one of these humors would disrupt the balance of these humors and cause disease [6]. “Abnormal black bile” is the term for an excess of black bile (literally “savda” in Arabic) and Melancholia or Depression was thought to be caused by “Abnormal black bile” by Galen which was adopted by Avicenna [7,8]. Although existence of black bile or abnormal black bile in body has never been confirmed in modern research, the demonstrated symptoms and signs of abnormal black bile were described in detail in Cannon of Medicine by Avicenna [9]. Although the four humors theory was dismantled from 19^th^ century, the definitive methods of Hippocratic medicine clinical diagnostics of abnormal humors have survived intact in the medical system of Uighur people in China. Traditional Uighur Medicine (TUM) considers the unbalanced humors or abnormal changes of humors are thought to cause disorders or diseases, and most clinical works identified the cause of complex diseases such as cancer, depression as an excess of black bile [10-12]., and some researches even indicate patients with “abnormal black bile” were significantly different from those with “non-abnormal black bile” (abnormal blood, yellow bile and phlegm) in terms of gene polymorphism, pre-thrombotic state and oxidative stress [13-15]. However, is “abnormal black bile” an existing phenomenon with distinct biological basis or merely imaginary philosophical concept that causes Depression? We undertook this investigation to answer this question.

The 5-HT_2A_ receptor is widely distributed in the central nervous system and periphery, and associated with major depressive illness [16]. Two single nucleotide polymorphisms, A-1438G and T102C are common in the population, show strong linkage disequilibrium with depression, and are associated with efficacy and positive side effect profiles of anti-depressant medications in some studies [17-20]. The 5-HT_1A_ receptor is a key mediator of serotonergic signaling in the central nervous system, and known as the major autoreceptor of serotonergic raphe neurons [21]. The C-1019G polymorphism of the 5-HT_1A_ gene promoter polymorphism affects 5-HT_1A_ receptor binding potential and protein expression [22]. Several studies suggest that GG genotype or G allele carriers of the 5-HT_1A_ gene promoter polymorphism have a greater risk of depression and poorer response to antidepressant therapy [18,22]. Brain-derived neurotrophic factor (BDNF) plays a critical role in neuronal growth, survival and differentiation neuronal cells of the central nervous system [23]. There is increasing evidence that the BDNF might be one of the relevant factors in the pathophysiology of depression [24]. Evidence of an association between the BDNF Val66Met polymorphism and depression is rapidly being replicated [25].

In the present study, we investigated the 5-HT_2A_ T102C, 5-HT_2A_ A-1438G, BDNF Val66Met and 5-HT_1A_ C-1019G gene polymorphisms in depression patients with two type of abnormal humor in TUM. Some of these gene polymorphisms have been thoroughly studied, but to our knowledge this is the first study to investigate the association of multiple polymorphisms and depression with abnormal humor in TUM. Our purpose was to investigate the association between some related gene polymorphisms and depression with abnormal humor, and whether certain genetic polymorphisms may be predictive markers of depression in patients with abnormal humor in TUM.

## Methods

### Patients and controls

#### Case selection

Patients including in this study met standards both of Western medicine and Traditional Uighur medicine. Specially, we followed the following criteria: (a) Uighur ethnic group, (b) aged 14 to 80 years, (c) meet the Mood disorder standard of Chinese Classification of Mental Disorders (CCMD-3), (d) the Hamilton Rating Scale for Depression (HAMD) 24-item version scale score ≥ 18, (e) patients or their parents gave informed consent for this study, and (f) there were no restrictions on gender.

We excluded patients: (a) with cerebrovascular disease or other serious organic brain diseases, (b) with high blood pressure, diabetes and other serious physical illness, (c) with other mental disorders associated with depression, (d) who has a history of manic episodes or anti-depressant induced mania, (e) who recently (within six weeks) received anti-depressant treatment.

#### Participates

We selected 201 patients of Uighur population who were diagnosed by two independent psychiatrists respectively (134 females and 67 males mean age 42.57 ± 15.64 years). While 50 healthy volunteers were used as controls (20 females and 30 males with a mean age of 28 ± 8.56 years). The research protocol was approved by the First Affiliated Hospital (First Affiliated Hospital, Xinjiang Medical University, Urumqi, China) ethics review committee. Written informed consent was obtained from each subject prior to the study.

#### Classification of depression by TUM

201 cases of depression patients were classified into abnormal black bile (ABB) group and none abnormal black bile (nABB) group according to humor classification criteria described in TUM [9,10]. The ABB group consisted of 72 females and 35 males with a mean age of 44.19 ± 15.43 years and with a mean HAMD score of 37.92 ± 11.53. The nABB group consisted of 62 females and 32 males with a mean age of 40.65 ± 16.68 years and with a mean HAMD score of 38.42 ± 13.59.

### DNA analysis and genotyping

#### DNA preparation

After 12 hours of fasting, 4 ml of venous blood was collected from each subject in 0.019 mol/L sodium citrate (1 portion anticoagulant + 9 portion whole blood). DNA was extracted from blood leukocytes with the TIANamp Blood DNA Kit (TIANGEN BIOTECH, Beijing).

#### 5-HT_2A_ genotyping

To genotype the A-1438G SNP in the 5-HT_2A_ gene, PCR was performed with the forward primer 5′-CTAGCCACCCTGAGCCTATG-3′ and the reverse primer 5′-TTGTGCAGATTCCCATTAAGG-3′. The amplification mixture contained 2 μl of 100 ng/μl DNA, 10 μl of 2 × Master Mix (2 × Taq PCR Master Mix, TIANGEN), 0.5 μl of 20 μM each primer, 7 μl of distilled water in a final volume of 20 μl. The amplification cycle was performed on a C1000™ Thermal Cycler (BIO-RAD, USA). After an initial denaturation at 94°C for 3 min, the DNA was amplified by 35 PCR cycles: denaturation at 94°C for 30 s, annealing at 58.1°C for 30 s and extension at 72°C for 1 min, followed by a final extension at 72°C for 7 min, and the reaction was terminated at 4°C. Amplified products were separated by electrophoresis on 1.5% agarose gel and visualized with ultraviolet light after ethidium bromide staining. The 2 × Master Mix contained 10 mM Tris–HCl (PH8.3), 100 mM KCl, 3 mM MgCl_2_, 500 μM dNTP each, 0.1 U Taq Polymerase/μl. The amplified DNA was digested at 37°C for 12 hours with the restriction enzyme MspI (Fermentas, USA), which cuts at the 1438G site. The product was electrophoresed on 3% agarose gels and stained with ethidium bromide. Homozygous genotypes were identified by the presence of a single 200-bp band (*AA*), or bands of 121- and 79-bp (*GG*). The heterozygous genotype (*AG*) had all three bands.

To genotype the T102C SNP in the 5-HT_2A_ gene, PCR was performed with the forward primer 5′-TCTGCTACAAGTTCTGGCTT-3′ and the reverse primer 5′-CTGCAGCTTTTTCTCTAGGG-3′ [26]. Amplification was performed using the same methods described for the 5-HT_2A_ A-1438G SNP. Allele 1 (T102-allele) was represented by the uncut 342-bp PCR product and allele 2 (C102-allele) consisted of two fragments at 217- and 125-bp.

#### BDNF genotyping

The genotyping of Val66Met polymorphism of the BDNF gene was determined by examining the occurrence of a *NlaIII* recognition site [27]. The PCR assay mixture contained 2 μl of 100 ng/μl DNA, 10 μl of 2 × Master Mix (2 × Taq PCR Master Mix, TIANGEN), 0.5 μl of 20 μM each primer (5′-ACTCTGGAGAGCGTGAAT-3′ and 5′-ATACTGTCACACACGCTC-3′) [28], and 7 μl of distilled water. The amplification cycle was performed on a C1000™ Thermal Cycler (BIO-RAD, USA). After an initial denaturation at 94°C for 5 min, the DNA was amplified by 35 cycles: denaturation at 94°C for 30 s, annealing 60°C for 30 s and extension 72°C for 1 min, followed by a final extension at 72°C for 7 min, the reaction was terminated at 4°C. PCR products were digested with *NlaIII* (Fermentas, USA) endonuclease, the product was electrophoresed on 3% agarose gels and stained with ethidium bromide. The presence of 168 and 75 bp bands indicates the existence of A (Met) allele; the presence of 243 bp band indicates the existence of G (Val) allele, while the presence of 75, 168 and 243 bp indicates AG (Met/Val) heterozygote.

#### 5-HT_1A_ genotyping

To genotype the C-1019G SNP in the 5-HT_1A_ gene, PCR was performed with the forward primer 5′-TGGAAGAAGACCGAGTGTGTCTAC-3′ and the reverse primer 5′-TTCTCCCTGAGGGAGTAAGGCTGG-3′ [29]. The amplification mixture contained 2 μl of 100 ng/μl DNA, 10 μl of 2 × Master Mix (2 × Taq PCR Master Mix, TIANGEN), 0.5 μl of 20 μM each primer and 7 μl of distilled water. Samples were amplified using a C1000™ Thermal Cycler (BIO-RAD, USA) for 36 cycles. After an initial 5 min at 95°C, each cycle consisted of 45 s at 95°C, 45 s at 56°C, and 45 s at 72°C. After a final 10 min at 72°C, the reaction was terminated at 4°C. The amplified DNA was digested with the restriction enzyme Hpy CH4IV (Fermentas, USA ), which cuts at the -1019G site, and the product was electrophoresed in 5% agarose gels and stained with ethidium bromide. Homozygous genotypes were identified by the presence of a single 182 bp band (C/C), or bands of 158 and 24 bp (G/G). The heterozygous genotype had three bands: 182, 158, and 24 bp (C/G).

### Statistical analysis

Data was analyzed using the statistical package for social sciences (SPSS ver.17). Quantitative variables were expressed as mean ± SD. Differences in variable means between ABB group and nABB group were compared by *T* test. Reported percentages reflect average values. Alleles, genotype frequencies, and individual features between patients and control subjects were compared by Pearson Chi-Square (χ^2^) and Continuity Correction Chi-Square test. Hardy-Weinberg equilibrium was assessed by Chi-Square analysis. A *P* value less than 0.05 was considered statistically significant.

## Results

### 5-HT_2A_ A-1438G polymorphism

**Figure 1:**
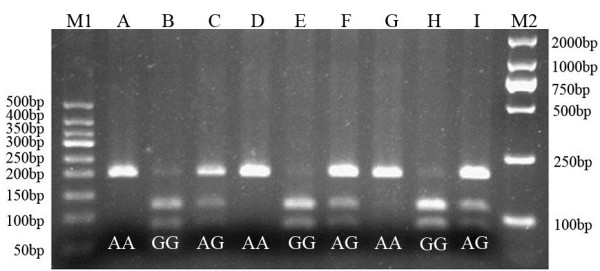
**Agarose gel eletrophoretogram of 5-HT**_**2A **_**A-1438G PCR product after digestion with MspI.** The distribution of the A-1438G polymorphism of 5-HT2A in nABB group and control group were in agreement with the Hardy-Weinberg equilibrium. There were no significant differences between groups (P > 0.05). Lane M1: 50 bp DNA Ladder; Lane A, D, and G: *AA*; Lane B, E, and H: *GG*; Lane C, F, and I: *AG*; Lane M2: 2000 bp DNA marker.

Homozygous genotypes were identified by the presence of a single 200-bp band (*AA*) or bands of 121- and 79-bp (*GG*). The heterozygous type (*AG*) displayed all three band sizes (Figure 1).

The distribution of the A-1438G polymorphism of 5-HT_2A_ in nABB group and control group were in agreement with the Hardy-Weinberg equilibrium. There were no significant differences between groups (*P* > 0.05). The Hardy-Weinberg equilibria for the candidate gene were as follows: nABB group, χ^2^= 0.095, df= 2, *P=* 0.954; control group, χ^2^= 0.665, df= 2, *P=* 0.717.

Frequencies of 5-HT_2A_ A-1438G alleles and genotypes in the ABB group, nABB group and control group are shown in Table 1. There were significant differences in the 5-HT_2A_ A-1438G genotype distributions among the ABB group,nABB group and control group (χ^2^= 13.310, *P=* 0.010). Compared with the control and nABB groups, the ABB group had significant differences (χ^2=^ 12.760, *P=* 0.002; χ^2^= 8.299, *P=* 0.016) in the A-1438G genotype distributions. Compared to control group, the nABB group had not a significant difference in the A-1438G genotype distributions (*P* > 0.05). Individuals with the *GG* (OR 0.176; 95% CI 0.057 ~ 0.538) genotypes were more likely to not have depression. Moreover, *GG* genotype seemed to be protective factor. There were no significant differences in the 5-HT_2A_ A-1438G allele distributions among the ABB group,nABB group and control group (χ^2^= 1.904, *P=* 0.386).

**Table 1:** 5-HT2A A-1438G, 5-HT2A T102C, BDNF Val66Met, and 5-HT1A C-1019G genotype and allele distribution in depression patients and controls

**Genotype and allele**	**ABB group (n= 106)**	**nABB group (n= 89)**	**Control group (n= 50)**	**χ2**	***P value***
	**n (%)**	**n (%)**	**n (%)**		
5-HT2A A-1438G					
Genotype AA	34(32.1)	29(32.6)	18(36.0)	13.31	0.01
*AG*	67(63.2)	45(50.6)	21(42.0)		
*GG*	5(4.7)	15(16.9)	11(22.0)		
*P value*	0.002^a^	0.59			
Alleles A	135(63.7)	103(57.9)	57(57.0)	1.9	0.39
*G*	77(36.3)	75(42.1)	43(43.0)		
*P value*	0.26	0.89			
5-HT2A T102C					
Genotype TT	34(32.1)	30(33.7)	19(38.0)	15.69	0
*TC*	68(64.2)	45(50.6)	20(40.0)		
*CC*	4(3.8)	14(15.7)	11(22.0)		
*P value*	0.000^b^	0.44			
Alleles T	136(64.2)	105(59.0)	58(58.0)	1.57	0.46
*C*	76(35.8)	73(41.0)	42(42.0)		
*P value*	0.3	0.87			
BDNF Val66Met					
Genotype Val/Val	51(48.1)	35(39.3)	12(24.0)	17.79	0
*Met/Val*	51(48.1)	42(47.2)	26(52.0)		
*Met/Met*	4(3.8)	12(13.5)	12(24.0)		
*P value*	0.040^c^	0.11			
Alleles Val	153(72.2)	112(63.0)	50(50.0)	14.77	0
*Met*	59(27.8)	66(37.0)	50(50.0)		
*P value*	0.000^d^	0.04			
5-HT1A C-1019G					
Genotype CC	46(43.4)	40(45.0)	15(30.0)	14.63	0.01
*CG*	55(51.9)	35(39.3)	23(46.0)		
*GG*	5(4.7)	14(15.7)	12(24.0)		
*P value*	0.001^e^	0.19			
Alleles C	147(69.3)	115(64.6)	53(53.0)	7.91	0.02
*G*	65(30.7)	63(35.4)	47(47.0)		
*P value*	0.005^f^	0.06			

^a^Odds ratio (and 95% confidence interval) for GG vs (A/G + A/A) is 0.176(0.057 ~ 0.538), χ2= 11.O25, df= 1, P= 0.001.

^b^Odds ratio (and 95% confidence interval) for CC vs (T/C + T/T) is 0.139(0.042 ~ 0.463), χ2= 12.987, df= 1, P= 0.000.

^c^Odds ratio (and 95% confidence interval) for Val/Val vs (Val/Met + Met/Met) is 2.936(1.383 ~ 6.232), χ2= 8.205, df= 1, P= 0.004, and Met/Met vs Val/Met + Val/Val is 0.124(0.038 ~ 0.409), χ2= 15.100, df= 1, P= 0.000.

^d^Odds ratio (and 95% confidence interval) for Met allele vs Val allele is 2.936(1.383 ~ 6.232), χ2= 14.692, df= 1, P= 0.000.

^e^Odds ratio (and 95% confidence interval) for GG vs (G/C + C/C) is 0.157(0.052 ~ 0.475), χ2= 13.010, df= 1, P= 0.000.

^f^Odds ratio (and 95% confidence interval) for C allele vs G allele is 2.006(1.229 ~ 3.271), χ2= 7.884, df= 1, P= 0.005.

### 5-HT_2A_ T102C polymorphism

**Figure 2:**
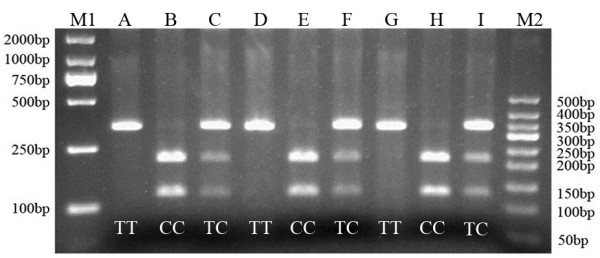
**Agarose gel eletrophoretogram of 5-HT**_**2A **_**T102C PCR product after digestion with MspI.** The distribution of the 5-HT_2A_ T102C polymorphism in nABB group and control group were in agreement with the Hardy-Weinberg equilibrium. There were no significant differences between groups (*P* > 0.05). Lane M1: 2000 bp DNA marker; Lane A, D, and G: *TT*; Lane B, E, and H: *CC*; Lane C, F, and I: *TC*; Lane M2: 50 bp DNA Ladder.

Homozygous genotypes were identified by the presence of a single 342-bp band (*TT*), or bands of 217- and 125-bp (*CC*). The heterozygous genotype (*TC*) had all three bands (Figure 2).

The distribution of the 5-HT_2A_ T102C polymorphism in nABB group and control group were in agreement with the Hardy-Weinberg equilibrium. There were no significant differences between groups (*P* > 0.05).The Hardy-Weinberg equilibria for the candidate gene were as follows: nABB group χ^2^= 0.096 df= 2 *P=* 0.953; control group χ^2^= 0.675 df= 2 *P=* 0.714.

Frequencies of the 5-HT_2A_ T102C alleles and genotypes in the ABB group, nABB group and control group are shown in Table 1. There were significant statistical differences in the 5-HT_2A_ T102C genotype distributions among the ABB group, nABB group, and control group (χ^2^= 15.686, *P=* 0.003). Compared with the control and nABB groups, the ABB group had significant differences (χ^2^= 15.602, *P=* 0.000; χ^2^= 9.074, *P=* 0.011) in the T102C genotype distributions. Compared to control group, the nABB group had not a significant difference (*P* > 0.05) in the T102C genotype distributions. Individuals with the *CC* (OR 0.139; 95% CI 0.042 ~ 0.463) genotypes were more likely to not have depression. Moreover, *CC* genotype seemed to be protective factor. There were no significant differences in the 5-HT_2A_ T102C allele distributions among the ABB,nABB and control group (χ^2^= 1.566, *P=* 0.457).

### BDNF Val66Met polymorphism

**Figure 3:**
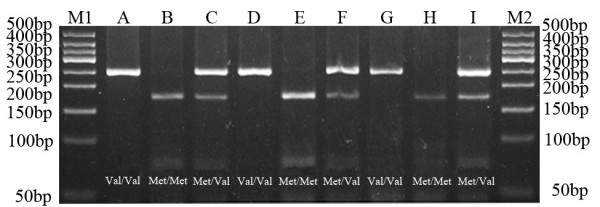
**Agarose gel eletrophoretogram of BDNF Val66Met PCR product after digestion with *NlaIII.*** The distribution of the BDNF Val66Met polymorphism in the ABB group, nABB group and control group were in agreement with the Hardy-Weinberg equilibrium. There were no significant differences between groups (*P* > 0.05). Lane M1: 50 bp DNA Ladder; Lane A, D, and G: *Val/Val*; Lane B, E, and H: *Met/Met*; Lane C, F, and I: *Met/Val*; Lane M2: 50 bp DNA Ladder.

Homozygous genotypes were identified by the presence of a single 243-bp band (*Val/Val*), or bands of 75- and 168-bp (*Met/Met*). The heterozygous genotype (*Met/Val*) had all three bands (Figure 3).

The distribution of the BDNF Val66Met polymorphism in the ABB group, nABB group and control group were in agreement with the Hardy-Weinberg equilibrium. There were no significant differences between groups (*P* > 0.05). The Hardy-Weinberg equilibria for the candidate gene were as follows: ABB group, χ^2^= 2.165,df= 2, *P=* 0.339; nABB group, χ^2^= 0.006,df= 2, *P=* 0.997; control group, χ^2^= 0.090,df= 2, *P=* 0.956.

The genotype and allele frequency distributions of BDNF Val66Met polymorphism in patients and control subjects is shown in Table 1. There were significant statistical differences in the BDNF Val66Met genotype distributions among the ABB group, nABB group, and control group (χ^2^= 17.793, *P=* 0.001). Compared with the control and nABB groups, the ABB group had significant differences (χ^2^= 6.414, *P=* 0.040; χ^2^= 18.547, *P=* 0.000) in the BDNF Val66Met genotype distributions. Compared to control group, the nABB group had not a significant difference (*P* > 0.05) in the Val66Met genotype distributions. Individuals with the *Met/Met* (OR 0.124; 95% CI 0.038 ~ 0.409) genotypes were more likely to not have depression. Moreover, *Met/Met* genotype seemed to be protective factor. In case of the genotypic association of BDNF, analyses using the recessive model showed association between the SNP and the risk of depression (χ^2^= 8.205,*P*= 0.004). Individuals with the *Met* allele more likely to be have depression (OR 2.936; 95% CI 1.383 ~ 6.232 for the recessive model). There were significant differences in the BDNF Val66Met allele distributions among the ABB,nABB and control group (χ^2^= 14.773, *P=* 0.001).

### 5-HT_1A_ C-1019G polymorphism

**Figure 4:**
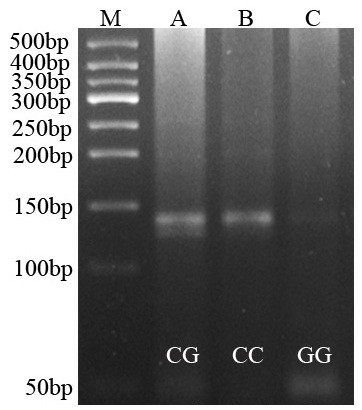
**Agarose gel eletrophoretogram of 5-HT**_**1A **_**C-1019G PCR product after digestion with Hpy CH4IV.** The distribution of the 5-HT_1A_ C-1019G polymorphism in the ABB group, nABB group and control group were in agreement with the Hardy-Weinberg equilibrium. There were no significant differences between groups (*P* > 0.05). Lane M: 50 bp DNA Ladder; Lane A: *CG*; Lane B: *CC*; Lane C: *GG*.

Homozygous genotypes were identified by the presence of a single 182-bp band (*C/C*), or bands of 24- and 158-bp (*G/G*). The heterozygous genotype (*C/G*) had all three bands (Figure 4).

The distribution of the 5-HT_1A_ C-1019G polymorphism in the ABB group, nABB group and control group were in agreement with the Hardy-Weinberg equilibrium. There were no significant differences between groups (*P* > 0.05). The Hardy-Weinberg equilibria for the candidate gene were as follows: ABB group, χ^2^= 2.924,df= 2, *P=* 0.232; nABB group, χ^2^= 0.951,df= 2, *P=* 0.622; control group, χ^2^= 0.161,df= 2, *P=* 0.923.

The genotype and allele frequency distribution of 5-HT_1A_ C-1019G polymorphism in patients and control subjects is shown in Table 1. There were significant statistical differences in the 5-HT_1A_ C-1019G genotype distributions among the ABB group, nABB group, and control group (χ^2^= 14.633, *P=* 0.006). Compared with the control and nABB groups, the ABB group had significant differences (χ^2^= 7.703, *P=* 0.021; χ^2^= 13.387, *P=* 0.001) in the 5-HT_1A_ C-1019G genotype distributions. Compared to the control group, the nABB group had not significant differences (P > 0.05) in the 5-HT_1A_ C-1019G genotype distributions. Individuals with the *GG* (OR 0.157; 95% CI 0.052 ~ 0.475) genotypes were more likely to not have depression. Moreover, *GG* genotype seemed to be protective factor. There were significant differences in the 5-HT_1A_ C-1019G allele distributions among the ABB,nABB and control group (χ^2^= 7.914, *P=* 0.019).

### Interaction analysis between the 5-HT_2A_ T102C and 5-HT_2A_ A-1438G polymorphisms in ABB patients and healthy volunteers

The two polymorphisms were in linkage disequilibrium. Table 2 shows that 93.3% of ABB and 78.0% of controls homozygous for 102 T were also homozygous for -1438A. In addition, 65.1% of ABB and 62.0% of controls homozygous for 102C were also homozygous for -1438G allele. The -1438A and 102 T allele frequencies were higher among ABB (0.64 and 0.64, respectively) than in controls (0.57 and 0.58, respectively). The OR associated with the presence of -1438A plus 102 T alleles was 3.989 for ABB compared with controls (OR 3.989; 95% CI 1.442 ~ 11.035; *P=* 0.005) (Table 2).

**Table 2:** Genotype distribution (n (%)) of T102C and A-1438G polymorphisms of the 5-HT2A gene among ABB patients and healthy volunteers

	**102 TT**	**102 TC**	**102 CC**
ABB group (n= 106)			
−1438 AA	31(29.2)	3(2.8)	
−1438 AG	3(2.8)	62(58.5)	2(1.9)
−1438 GG		3(2.8)	2(1.9)
Control group (n= 50)			
−1438 AA	18(36.0)		
−1438 AG	1(2.0)	20(40.0)	
−1438 GG			11(22.0)

### Interaction analysis between the BDNF Val66Met and 5-HT_1A_ C-1019G polymorphisms in ABB patients and healthy volunteers

Significant difference was observed between the ABB and control groups (χ^2^= 14.692,*P*= 0.000) in the BDNF Val66Met allele distribution. The *Val* allele was associated with 2.593 fold increased risk for depression compared with *Met* allele (OR 2.593; 95% CI 1.583 ~ 4.249). Moreover, there was a significant difference in the 5-HT_1A_ C-1019G allele distribution between the ABB and control groups (χ^2^= 7.884,*P*= 0.005). The *C* allele was associated with 2.006 fold increased risk for depression compared with *G* allele (OR 2.006; 95% CI 1.229 ~ 3.271).

The OR associated with the combination of Val66Met-Val/Val genotype plus the presence of -1019C allele was 8.393 for ABB compared with controls (OR 8.393; 95% CI 1.807 ~ 38.991; *P=* 0.003). Moreover, the OR associated with the presence of -*Met* plus -1019C alleles was 12.194 for ABB compared with controls (OR 12.194; 95% CI 1.433 ~ 103.776; *P=* 0.005). The OR associated with the presence of -1438*C/C* plus *Val/Val* genotypes was 7.738 for ABB compared with controls (OR 7.738; 95% CI 1.566 ~ 38.241; *P=* 0.005) (Table 3).

**Table 3:** BDNF Val66Met (5-HT1A C-1019G) genotype frequency in ABB patients and healthy volunteers according to 5-HT1A C-1019G (BDNF Val66Met) genotype

**Genotypes**		**Genotype frequency (n (%))**		***P value***	**OR(95% CI)**
		**ABB group (n= 106)**	**Control group (n= 50)**		
Val66Met	C-1019G				
*Val/Val*	*CC*	25(49.0)	2(16.7)	0	1
	*CG*	22(43.2)	5(41.7)		
	*GG*	4(7.8)	5(41.7)		8.393(1.807 ~ 38.991)
*Met/Val + Met/Met*	*CC*	21(38.2)	13(34.2)	0.01	1
	*CG*	33(60.0)	18(47.4)		
	*GG*	1(1.8)	7(18.4)		12.194(1.433 ~ 103.776)
C-1019G	Val66Met				
*CC*	*Val/Val*	25(54.3)	2(13.3)	0.01	1
	*Met/Val + Met/Met*	21(45.7)	13(86.7)		7.738(1.566 ~ 38.241)
*CG*	*Val/Val*	22(40.0)	5(21.7)	0.12	1
	*Met/Val + Met/Met*	33(60.0)	18(78.3)		2.400(0.777 ~ 7.416)
*GG*	*Val/Val*	4(80.0)	5(41.7)	0.15	1
	*Met/Val + Met/Met*	1(20.0)	7(58.3)		5.600(0.472 ~ 66.447)

### Identification of independent risk factors for ABB

Table 4 presents the estimated logistic regression coefficients, estimated SEs, adjusted odds ratios (ORs), and 95% confidence intervals (CIs) for the adjusted ORs for the final model for admissions. After adjusting for those conventional depression risk factors such as age, gender, the *AA* (of the A-1438G polymorphisms of the 5-HT_2A_ gene), *Val/Val* (of the Val66Met polymorphisms of the BDNF gene), *CC* (of the C-1019G polymorphisms of the 5-HT_1A_ gene) genotype still had an approximately. A multiple logistic regression model showed 5 independent factors: Gender (OR= 0.365, 95% CI: 0.136-0.982; *P* < 0.05), Age (OR= 4.806, 95% CI: 2.476-9.328; *P* < 0.01), *AA* (OR=13.759, 95% CI: 2.225-85.076; *P* <005; for 5-HT_2A_ A-1438G), *Val/Val* (OR= 39.791, 95% CI: 5.365-295.110; *P* <0.01), *CC* (OR= 7.309, 95% CI: 1.349-39.597; *P* <0.05; for 5-HT_1A_ C-1019G). In addition, we found *Val/Met* genotype of the BDNF Val66Met gene is also an independent risk factor for ABB with an odds ratio of 6.854-fold higher relative risk of developing ABB than those with the *Met/Met* homozygote (OR= 6.854, 95% CI: 1.092-43.033; *P* < 0.05), also *AG* (OR=32.889, 95% CI: 4.981-217.178; *P* < 0.01; for 5-HT_2A_ A-1438G) and *CG* (OR= 7.420, 95% CI: 1.282-42.965; *P* < 0.05; for 5-HT_1A_ C-1019G) genotypes are an independent risk factor for ABB.

**Table 4:** Results of logistic analysis

	**ABB**					
	**B**	**S.E.**	**Wald**	***P***	**OR**	**95% C.I.**
Gender	−1.01	0.51	3.98	0.05	0.37	0.136-0.982
Age	1.57	0.34	21.52	0	4.81	2.476-9.328
A1438G(GG)			13.36	0		
A1438G(AA)	2.62	0.93	7.95	0.01	13.76	2.225-85.076
A1438G(AG)	3.49	0.96	13.16	0	32.89	4.981-217.178
BDNF(Met/Met)			15.37	0		
BDNF(Val/Val)	3.68	1.02	12.98	0	39.79	5.365-295.110
BDNF(Val/Met)	1.93	0.94	4.22	0.04	6.85	1.092-43.033
5-HT1A(GG)			5.74	0.06		
5-HT1A(CC)	1.99	0.86	5.32	0.02	7.31	1.349-39.597
5-HT1A(CG)	2	0.9	5	0.03	7.42	1.282-42.965
Constant	−7.28	1.66	19.21	0	0	

## Discussion

Traditional Uighur Medicine (TUM), a well-known medical system in China for their remarkable curative effect, is based on the concept of equilibrium and balance of natural body humors (blood, phlegm, yellow bile, and black bile). The imbalance in the quality and quantity of these humors leads to diseases whereas restoration of this balance maintains health of a person [10]. Abnormal humors include abnormal blood, abnormal phlegm, abnormal yellow bile and abnormal black bile. According to TUM, abnormal black bile is a special humor in the body, and is the main cause of complex diseases such as tumors, diabetes mellitus, hypertension, and depression [12].

In this study, we evaluated the individual and interaction effects of depression related gene polymorphisms in depression with abnormal humor. Our results indicated that there were significant interactions of the T102C and A1438G SNPs in 5-HT_2A_ receptor gene, Val66Met SNP in BDNF gene, and C-1019G SNP in 5-HT_1A_ receptor gene with depression with abnormal humor. Moreover, the present study showed a significant interaction between the BDNF Val66Met and 5-HT_1A_ C-1019G polymorphisms.

The present study on ABB revealed significant difference in the prevalence of 5-HT_2A_ T102C and 5-HT_2A_ A-1438G genotype frequency compared with that of controls and nABB patients. These results reported here in a Chinese population were not in agreement with those reported by Dawei Li, who found no association of the T102C polymorphism with either schizophrenia or suicidal behavior, evidence of significant association was only detected between the A-1438G polymorphism and suicidal behavior [30]. Our results failed to confirm previous reports that genotype frequencies of 102 T/C 5-HT_2A_ receptor gene polymorphism in mood disorders do not differ from healthy volunteers [31-33]. Moreover, Ho-kyoung Yoon concluded that the 5-HT_2A_ T102C polymorphism may not be associated with susceptibility to suicidal behavior in Korean population [34]. We postulate that the differences in the genotype frequencies between our study and previous reports may be the result of ethnic differences of patients participated in these studies. Furthermore, no difference was found in 5-HT_2A_ T102C genotype and allele distribution between the mood disordered subjects, with and without suicide attempt history, and controls [35]. The authors concluded that their study did not support the association of the 5-HT_2A_ gene with either schizophrenia or suicidal behavior. The results showed a higher frequency of 5-HT_2A_ –1438A and 102 T alleles in ABB compared to controls. We found that the 1438G, 102 T alleles and the homozygosity for these alleles were significantly more frequent among the patients with ABB than among the controls, suggesting that the allele is associated with susceptibility to depression. We also found that *AA* (or *TT*) genotype is an independent risk factor for ABB according to the multiple logistic regression models.

In this study, we found a significant difference among the *Val/Val*, *Val/Met*, and *Met/Met* genotypes or between *Val* and *Met* alleles in ABB group patients compared with the controls and nABB patients. Our results reported here in a Chinese population are in agreement with those reports such as BDNF may have a major role in the pathogenesis and treatment response of depression [36]. Two meta-analyses have suggested that BDNF is associated with depression or the response to antidepressants [37,38], while another meta-analysis showed no association [39]. In this study, ABB individuals with the major allele (*Val/Val*) had an odds ratio of 2.9, also *Val/Val* genotype is an independent risk factor for ABB according to the multiple logistic regression models. Our results confirmed previous reports that BDNF polymorphism is associated with increased risk of depression in some studies [40,41], as some studies found no association [18,42-44]. In our study *Val* allele has a higher distribution in the Chinese population, and *Val/Val* genotype or carrying Val allele of BDNF Val66Met polymorphism is associated with higher response to depression. Approximately 30%-50% of people worldwide are either heterozygous (*Val/Met*) or homozygous (*Met/Met*) for the methionine substitution [45]. Although there is a wealth of information about individuals heterozygous for the Met polymorphism, little information exists about individuals who are homozygous for the Met allele (*Met/Met*), because this genotype is rare in the general population, comprising only 4% of people in the United States [45]. In studies of brain morphometry using structural MRI scans, *Val/Met* individuals have repeatedly been shown to have a smaller hippocampal volume relative to controls to who are homozygous for the *Val* allele (*Val/Val*) [46,47]. This difference may be related to the role BDNF and its receptors play in the development as well as continued plasticity of the brain [48,49].

In this study, we found a significant difference between 5-HT_1A_ gene *CC*, *CG*, and *GG* genotype or between *C* and *G* allele in ABB group patients compared with the controls and nABB patients. We found that the *CC* allele and the homozygosity for this allele were significantly more frequent among the patients with ABB than among the controls, suggesting that the allele is associated with susceptibility to depression. Our results were not in agreement with these reports that *GG* genotype is associated with increased risk of depression [22] and suicide [21]. In our study *C* allele has a higher distribution in the Chinese population, and *CC* genotype or carrying *C* allele of 5-HT_1A_ C1019G gene is associated with higher response to depression patients with abnormal humor syndrome. We also found that ABB individuals with the major allele (*CC*) had an odd ratio of 1.8; *CC* genotype is an independent risk factor for ABB according to the multiple logistic regression models.

In the present study, subjects carrying both 5-HT_1A_ C1019G polymorphism *CC* genotype and *Val/Val* genotype of BDNF Val66Met polymorphism had over seven times higher risk of depression than those who did not have this combination of polymorphisms. We reported a significant association between combined polymorphisms of 5-HT_1A_ and BDNF polymorphisms and our result was in line with earlier reports [50].

In summary, the present study demonstrated that the A-1438 and T102C polymorphisms of the 5-HT_2A_ receptor gene, BDNF Val66Met, and 5-HT_1A_ C-1019G might predict the incidence of depression induced by abnormal black bile. The results showed a higher frequency of 5-HT_2A_ –1438A and 102 T alleles. 5-HT_2A_ –1438A and 102 T polymorphisms were in linkage disequilibrium. ABB individuals with the major allele (*Val/Val*) had a three times of odds ratio, also ABB individuals with the major allele (*CC*) had a two times of odds ratio. The *AA*, *Val/Val, and CC* genotypes were independent risk factors for the ABB. There was a significant association between combined polymorphisms of 5-HT_1A_ and BDNF polymorphisms. However, it was the first study to investigate the association of multiple gene polymorphisms and depression with abnormal humor in TUM. Of course, as an ancient medical system, principles of TUM classification of depression contain a variety of biological foundation. But the present study at least to provide new ideas for individualized diagnosis, prevention, treatment of depression.

## Conclusions

It was concluded that there were significant relationship between the gene polymorphisms and classification of depression with abnormal humor in TUM. The 5-HT2A A-1438G, 5-HT2A T102C, BDNF Val66Met, and 5-HT1A C-1019G gene polymorphisms might predict the incidence of depression with ABB. It was the first study to investigate the association of multiple gene polymorphisms and depression with abnormal humor in TUM. Of course, as an ancient medical system, principles of TUM classification of depression contain a variety of biological foundation. But the present study at least to provide new ideas for individualized diagnosis, prevention, treatment of depression.

## Abbreviations

TUM: Traditional Uighur medicine; ABB: Abnormal black bile; nABB: None abnormal black bile; PCR-RFLP: Polymerase chain reaction-restriction fragment length polymorphism; SNPs: Single nucleotide polymorphisms; 5-HT2A: Serotonin 2A; 5-HT1A: Serotonin 1A; BDNF: Brain derived neurotrophic factor; CCMD-3: Mood disorder standard of Chinese classification of mental disorders; HAMD: Hamilton rating scale for depression.

## Competing interests

The authors declare that they have no competing interests.

## Authors’ contributions

AY designed the study. HU performed genotyping, made statistical analysis, managed literature search, interpreted the data and wrote the manuscript. AA collected samples. HU (corresponding author) gave comments to the manuscript. All authors read and approved the final manuscript.

## Pre-publication history

The pre-publication history for this paper can be accessed here:

http://www.biomedcentral.com/1472-6882/13/332/prepub
